# Man's Wants

**Published:** 1890-02-15

**Authors:** 


### Words of Consolation.

### MAN'S WANTS.

*(For Reading to the Sick.)*

We have seen in the case of the Prodigal Son how discontented is the heart of man while he is wandering from God, and that until he is reconciled to his Maker he can never find rest or peace. The very depths of the Prodigal's misery led him back to his father's embrace, but the generality of men go on in a blind way groping after happiness which, if they could only see, they would find within their grasp. "They look before and after, and pine for what is not." They say with the Eastern poet:

> We are the voices of the wandering wind,
> Which moan for rest, and rest can never find.
> Lo! as the wind is, so is mortal life—
> A moan, a sigh, a sob, a storm, a strife.

If this were a true description of mortal life, it would indeed be sad and full of suffering; but we have a "larger hope," and it only requires us to *know what* we want for our needs to be supplied. Let us try to find out what these wants are which stand in the way of our happiness. We want riches? Undoubtedly; but not the wealth of this world—not gold and jewels, nor the stuffs which perish in the using, but the Pearl of great price, the Wisdom far above rubies, the Treasures of Heaven where neither moth nor rust doth corrupt, and where thieves do not break through nor steal. If our treasure be there, there will our hearts be also, and in them will they find rest.

Do we think learning and knowledge will suffice us if we can drink our fill? Solomon, whose "wisdom exceeded all the wisdom of the East," and to whom kings came to listen, declares that he that increaseth knowledge increaseth sorrow.

Alas! for her who sets her heart on fine clothes, who barters her good name, her purity perhaps, her hopes in this world and the next, for the sake of admiration. Poor soul! she has thrown away the real adornment of a meek and quiet spirit.

What we want one and all is a new heart, a heart to love and fear God. Seek for it with a full determination to get what you ask for, for this alone will ensure our happiness. The unjust judge gave the poor widow what she required because of her importunity, and will not God hear the prayers of those who call upon Him day and night?

"Create in me a clean heart, O God, and renew a right spirit within me," is the cry of the penitent. Let us say the same. We cannot go on in our wanderings from the fold of Christ, we are so sad, so miserable. O, my Saviour, all is restless and unquiet out of Thee! Perform, we pray Thee, Thy gracious promises. "Thou wilt keep him in perfect peace whose mind is stayed on Thee, because he trusteth in Thee."

